# Comparison of the diagnostic performance of microscopic examination, Copro-ELISA, and Copro-PCR in the diagnosis of *Capillaria philippinensis* infections

**DOI:** 10.1371/journal.pone.0234746

**Published:** 2020-06-17

**Authors:** Mervat M. Khalifa, Salma M. Abdel-Rahman, Hanaa Y. Bakir, Ragaa A. Othman, Mohamed A. El-Mokhtar

**Affiliations:** 1 Medical Parasitology Department, Faculty of Medicine, Assiut University, Assiut, Egypt; 2 Medical Microbiology and Immunology Department, Faculty of Medicine, Assiut University, Assiut, Egypt; Beni Suef University, Faculty of Veterinary Medicine, EGYPT

## Abstract

Intestinal capillariasis is a parasitic zoonosis caused by the tiny nematode parasite *Capillaria philippinensis*. It is a major health problem that may lead to death if not diagnosed and treated appropriately. The difficulties in the diagnosis of *C*. *philippinensis* highlight the importance of developing accurate, sensitive, and specific methods for early diagnosis. This study aimed to detect the presence of *C*. *philippinensis* infection among 42 clinically suspected patients with certain criteria that are highly suggestive of capillariasis and to compare the diagnostic yield of microscopy, copro-ELISA, and PCR for the detection of copro-DNA. Sociodemographic characteristics and clinical data were also described for the infected group. Out of 42 patients, 10 were microscopically positive, 40 samples were positive by copro-ELISA, nested PCR detected 35 positive cases, with total detection rates of 23.8%, 95.2%, and 83.3% using direct microscopic examination, copro-ELISA, and PCR, respectively. The majority of positive cases were females, middle-aged people, and people from rural areas. The real number of cases infected with *C*. *philippinensis* may far exceed those estimated using microscopy. The diagnosis by copro-ELISA for the detection of *C*. *philippinensis* coproantigen and by nested PCR to identify parasite DNA revealed a higher number of positive cases. Using ELISA for the detection of coproantigen is a sensitive test that identifies the infection, yet it is not specific. Copro-DNA offers a satisfactory sensitive and specific method for the detection of infection in clinically suspected patients. The most susceptible individuals to *C*. *philippinensis* infection are females, middle-aged people, and people of low social standards. Intestinal capillariasis needs to be considered in patients who present with symptoms of chronic diarrhea and hypoalbuminemia because if these cases are left undiagnosed and untreated, they may suffer from lethal complications.

## Introduction

Intestinal capillariasis is an emerging zoonotic parasitic disease caused by the tiny nematode *Capillaria philippinensis*, which is related to fish. It is an important human parasitic infection primarily because of its potential for serious and even lethal complications in untreated patients. The parasite is also characterized by its ability to auto-infect the host, producing hyper infection **[1]**.

*C*. *philippinensis* parasite burden may increase to very high levels in the small intestine and may result in massive small bowel dysfunction, abdominal pain, massive chronic diarrhea, borborygmus, malabsorption, water and electrolyte imbalance, and marked weight loss. Infected individuals may become ill for months without a suitable anthelminthic chemotherapy because of the misdiagnosis. Death may occur because of the irreversible effects of electrolyte loss, resulting in heart failure or septicemia, which may result from a secondary bacterial infection **[2].**

Capillariasis was first reported in the Philippines **[3],** followed by reports of sporadic cases in other areas **[4].** More than 2,000 cases of intestinal capillariasis have been reported in the Philippines and Thailand, with sporadic cases reported in Korea, Japan, Taiwan, India, Iran, Egypt, Italy, the United Arab Emirates, Spain, and the United Kingdom **[1]**. Small freshwater and brackish-water fish are the sources of infection, and fish-eating birds are the reservoir hosts **[4]**. In Egypt, it was first reported by **Youssef et al. (1989) [5]**. Since then, many cases have been diagnosed in different areas of Egypt **[6]**.

The conventional diagnosis of intestinal capillariasis relies on the detection of characteristic peanut-shaped parasitic eggs with flattened bipolar plugs and a striated shell **[7]**. The accuracy of the conventional stool analysis is low, as patients can be misdiagnosed by inexperienced parasitologists, because of the scarcity of worm eggs in stool specimens or misdiagnosis with other trichurid eggs **[8]**.

There have been few previous studies on the immunodiagnosis of intestinal capillariasis owing to difficulties in obtaining the antigen of *C*. *philippinensis*. Therefore, some previous researchers used *Trichinella spiralis* antigen **[2, 8, 9]** for the immunodiagnosis of *C*. *philippinensis* infection. Others evaluated the diagnosis of *C*. *philippinensis* infection by the detection of coproantigen **[10]**. Coproantigen ELISA was used for the detection of some intestinal nematode infections, including *Strongyloides stercoralis* **[11]**. PCR, which is a technique based on detecting the DNA of the parasite, is valuable in the diagnosis of even low-intensity infections **[12]** and may be useful in enhancing the accuracy of the diagnosis of this disease **[13]**.

This study aimed to highlight the importance of different diagnostic methods for the early and accurate detection of *C*. *philippinensis* infection. We compared the ability of three diagnostic techniques–coproscopy, copro-ELISA, and copro-DNA to detect *C*. *philippinensis* in patients with clinical criteria that are highly suggestive of capillariasis. We also correlated the sociodemographic characteristics, clinical data, and microscopy, ELISA, and PCR results.

## Materials and methods

### Ethics statement

Written informed consent was obtained from all patients (adults and parents or guardians of children participants) before enrollment. The research protocol was approved by the ethics committee of the Faculty of Medicine, Assiut University.

Animal experiments were performed in the Animal House facility, Faculty of Medicine, Assiut University. The research protocol was approved by the Animal House ethics committee, Faculty of Medicine, Assiut University (application No. 17200263). Animal handling protocols conformed to the recommendations of the National Institutes of Health’s Guide for the Care and Use of Laboratory Animals used in other Egyptian universities and research centers.

### Study area and population

This study was conducted in the research laboratory of the Parasitology Department at the Faculty of Medicine, Assiut University. This study was conducted on 42 out of 800 patients who presented with chronic diarrhea and were admitted to the Internal, Tropical, or Pediatric Departments at Assiut University Hospital from May 2016 to February 2018. With the exclusion of patients with other causes of chronic diarrhea such as irritable bowel syndrome, viral and bacterial infections, and malignancy, only these 42 patients presented certain clinical and investigative criteria suggestive of *C*. *philippinensis* infection according to **Intapan et al. (2006) and Ali et al. (2016) [2, 14]**. These criteria included massive chronic diarrhea (ranging from 1–24 months) with hypokalemia and hypoalbuminemia and one or more of the following complaints: abdominal pain, borborygmi, weight loss (up to 30 kg with observed muscle wasting, profound weakness, and fatigue), or edema of the lower limb. Full patient history was obtained from each patient or the patient’s relatives (including age, sex, address, occupation, food habits, duration of the illness, family history, and traveling history). Physical examination, chest radiography, and abdominal ultrasonography were performed. Laboratory investigations were conducted on samples obtained from all patients, including urine analysis, complete blood count, serum albumin, and electrolyte analysis. The patients' fresh stool samples were sent to the department’s laboratory for further analysis. Ten clinically healthy persons were included as negative controls.

### Parasitological analysis

Fecal samples (2–5 g of fresh stool) were examined macroscopically for consistency, color, and presence of blood and/or mucus. Direct smear examination and formalin-ether concentration techniques were performed for each stool sample to detect the presence of *C*. *philippinensis* eggs, larvae, and/or adults. The stool analysis was repeated three times over 1- to 2-day intervals if the first examination was negative. Part of the fecal specimen was processed for the detection of coproantigen and copro-DNA.

#### Direct smear

Two preparations were made from each stool specimen by placing a drop of saline on the slide and a drop of 1% Lugol's iodine on another slide. Next, 4 mg of the stool was placed on the slide and a homogeneous thin film was prepared by mixing the stool with a drop of normal saline or 1% iodine. A cover glass was placed on each preparation and examined systematically using the low powers of the microscope **[15]**.

#### Formalin-ethyl acetate sedimentation concentration method [16]

One gram of stool was transferred to a glass beaker and emulsified in formalin (10%) using a glass rod, strained through 2 layers of wet gauze inside a funnel into a 15 mL glass conical centrifuge tube, and centrifuged at 1500 rpm for 5 min. The supernatant was discarded and 5 mL formalin (10%) was added and shaken well. Next, 3 mL of ether was added, and the tube was covered firmly with a rubber stopper. After that, the tube was shaken vigorously for 1 min and centrifuged at 1500 rpm for 5 min. Four separate layers were obtained: top layer ether, a plug of fecal debris, formalin, and at the bottom the sediment. The plug of debris was loosened from the sides of the tube with a stick, and all the supernatant fluid was decanted. The remaining sediment was examined using stained and unstained direct smears.

Each patient was administered a single dose of levamisole (Ketrax, 2.5 mg/kg), which is an anthelmintic drug that causes paralysis in the muscles of adult worms to mediate the expulsion of intact adult worms in the stool **[17].** After 24 h of drug administration, stool samples were collected and examined, and the expelled worms were isolated using pasture pipettes. Regarding the anthelmintic treatment regimen, adults received albendazole at a dose of 200 mg/day twice a day for three weeks, and children were given a dose of 100 mg/day. Patients were followed up by examining stool samples every month for 6 months.

### Preparation of the coproantigen

Stool elutes from the 42 patients and 10 healthy persons (negative control) were prepared according to the method of **Sykes and McCarthy (2011) [11]** by adding three parts of 0.01 M phosphate-buffered saline (PBS) containing 0.05% Tween 20 (PBST) to one part of the stool in a centrifuge tube. This mixture was then homogenized for 3 min, sonicated for 5 min in an ice bath, and then centrifuged at 10,000 rpm for 10 min. The supernatant was aspirated and the protein content was estimated, aliquoted, and kept frozen at 70°C until use.

### Preparation of *C. philippinensis* crude worm antigens

The crude worm antigen was prepared as described by **El-Dib et.al. (2004) [10]** with modifications. Briefly, adult worms were isolated from fresh stool samples of infected patients. A drop of liquid stool was placed in small Petri dishes containing a small amount of PBS (pH 7.4) and then examined using an inverted microscope. Using a micropipette, the tiny worms were transferred to small Eppendorf tubes containing 0.5 mL PBS (pH 7.4). Worms were washed three times in PBS, homogenized in a suitable amount of buffer, and then centrifuged at 6000 rpm for 3 min. The supernatant was aspirated and its protein content was estimated using a NanoDrop Spectrophotometer, then aliquoted and stored at –70°C until use.

### Preparation of hyperimmune sera against the crude worm antigen

Rabbit anti*-C*. *philippinensis* was obtained by inoculating two female New Zealand white rabbits with crude worm antigen of *C*. *philippinensis* according to the method of **Langley and Hillyer (1989) [18]** with modification. Primary dose of the crude worm antigen, adjusted to contain 1.2 mg protein and mixed in an equal volume of Freund's complete adjuvant, was injected subcutaneously into several points on the back of the rabbits. The rabbits were given similar inoculations on three dates with one-week intervals, but incomplete Freund's was used as an adjuvant. Similarly, rat anti-*C*. *philippinensis* was obtained by inoculating two white albino rats with one-third of the previous dose of the antigen. Blood was drawn 7–15 days after the final injection. The serum was pooled and stored at 20 ºC. Hyperimmune sera were evaluated using indirect ELISA against the crude *C*. *philippinensis* worm antigen and available helminth antigens in our department (crude hydatid fluid (CHF) antigen, *Schistosoma mansoni* egg antigen) **[19]**.

### Detection of *C. philippinensis* coproantigen using sandwich ELISA

*C*. *philippinensis* coproantigen was detected using sandwich ELISA as described by **Abdel-Rahman et al. (1998) [20]** with some modifications. The optimal rat and rabbit anti- *C*. *philippinensis* hyperimmune sera dilutions were 1:100 and 1:200, respectively, as determined by checkerboard titration. Polystyrene microtiter plate wells were sensitized overnight at 4 ºC with 100 μL/well of rat anti-*Capillaria* hyperimmune serum at a 1:100 dilution in carbonate buffer, pH 9.6. After washing three times with PBS containing 0.1% Tween (PBS/T), the unbound sites were blocked with 200 μL/well of 3% bovine serum albumin (BSA; Sigma) in PBS/T, pH 7.4. After 2 h of incubation at room temperature, the wells were washed. Stool supernatants were diluted 1:2 in 1% BSA in PBS/T and 100 μL dilution buffer and added to each well. The plate was incubated for 1 h at 4°C and then washed as described above. Subsequently, 100 μL/well of rabbit anti-*Capillaria* hyperimmune serum diluted 1:200 in dilution buffer was added to each well, then incubated for 1 h at room temperature. The wells were washed and 100 μL of peroxidase-conjugated goat anti-rabbit IgG (Santa Cruz) diluted 1:1000 in dilution buffer was added and incubated for 1 h. Then, the wells were washed three times, and 100 μL of 3,3,5,5-tetramethylbenzidine (TMB) (Santa Cruz) substrate was added. After 15 min, the reaction was stopped by adding 50 μL of 0.1 M sulfuric acid. The color intensity was measured at 450 nm using a microplate reader. The crude worm antigen, negative stool supernatant, and blocking solution were always included as controls.

### Detection of copro-DNA using nested PCR

Fecal samples were subjected to genomic DNA extraction using the QIAamp DNA Stool Mini Kit (Qiagen) following the manufacturer’s instructions with some modification in the thermal treatment of samples. The incubation period of the sample was prolonged from 1 h to overnight at 56°C, followed by incubation at 95°C for 1 h.

DNA amplification of the small subunit ribosomal DNA (ssurDNA) gene sequence was performed according to the method of **El-Dib et al. (2015) [13]**. The first reaction incorporated the CAP18SForw-1 and CAP18SRev-1 primers, while in the second, CAP18SForw-2 and CAP18SRev-2 were used. In the first PCR, a 235 bp fragment was expected, while in the second PCR, a 183 bp fragment was expected. PCR reactions were carried out using a Veriti™ 96-well thermal cycler (Applied Biosystems, USA). PCR components in both reactions consisted of 75 mM Tris-HCl (pH 9), 2 mM MgCl_2_, 50 mM KCl, 20 mM (NH_4_)_2_ SO_4_, 200 μM each of dNTPs, and the PCR primers, 1.25 U or 0.75 U DNA Taq polymerase (1 U/μL) (Biotools, Spain) for the first and second PCR reactions, respectively, in a final volume of 25 μL. Five microliters of the purified genomic DNA was used as templates in the first PCR reaction. For the second reaction, 2 μL of the PCR product of the first run was used as a template. PCR conditions included an initial denaturation step for 7 min at 94°C, followed by 40 cycles of 15 s at 94°C, 15 s at 58°C, and 30 s at 72°C. The second reaction consisted of 35 cycles; each cycle consisted of a denaturation step for 15 s at 94°C, 15 s at 62°C, and 20 s at 72°C, after which a final extension step of 10 min at 72°C was employed. Each amplification run contained a negative control (DNAase-free water and negative tissue) and positive control. After electrophoresis separation on 2% agarose gel, a visual inspection of the amplified products was performed using ethidium bromide staining.

### Statistical analysis

The collected data and the results were analyzed using SPSS, version 16. Frequency, cross-tabulation, chi-square with Fisher’s exact test, and the existing association were investigated. Values of P < 0.05 were considered statistically significant.

## Results

### Analysis of patient demographics and clinical data

As described in Table 1, the majority of patients 33/42 (78.6%) were females (these 42 patients were selected from 800 patients with chronic diarrhea, according to the previously mentioned clinical criteria suggestive for *C*. *philippinensis*). The most common age group was between 20 and 45 years (61.9%). Most of the female patients (n = 30) were housewives. Out of 9 male cases, 2 were fishermen. The majority of patients lived in rural areas 31 (73.8%). The 42 cases presented clinical manifestations suggestive of intestinal capillariasis infection. Regarding the possible risk factors of intestinal capillariasis, the majority of patients (90.5%) had eviscerated fish by themselves.

**Table 1:** Demographic characteristics and clinical data of the study groups.

	Total No. (n = 42)	(%)
**Age groups:**		
< 20 years	10	23.8
20–45 years	26	61.9
> 45	6	14.3
Mean: 31.8 ± 15.5 (4–68)		
**Sex:**		
Female	33	78.6
Male	9	21.4
**Residence:**		
Rural	31	73.8
Urban	11	26.2
**Diarrhea:**	42	100%
**Duration of diarrhea**		
1:4 month	25	59.5
> 4 month	17	40.5
**Borborygmi**	28	66.7
**Abdominal pain**	23	54.6
**Weight loss**	31	73.8
**Lower limb edema**	19	54.2
**Vomiting**	18	42.9
**Low-grade fever**	13	31

### Parasitological analysis

**Fig 1:**
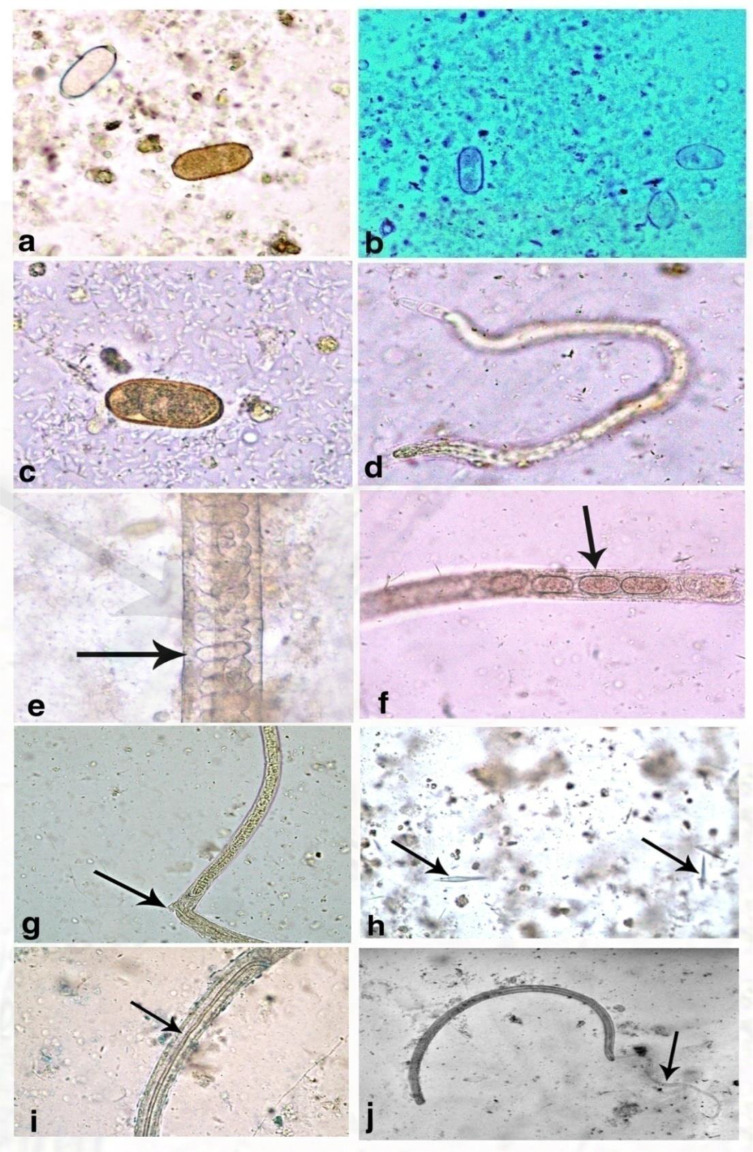
Microscopic examination of *C*. *philippinensis* showing different stages. A) Thick and thin-shelled egg; b) Swollen egg; c) Emberyonated egg; d) Sheathed larva; e) Adult female anterior end showing esophagus with stichocytes; f) adult female uterus containing eggs; g) Vulva of an adult female; h) Charcot–Leyden crystals; i) Posterior end of adult male showing transparent sheath containing spicule; j) Posterior end of adult male with projecting spicule.

Microscopic examination of the stool was performed using direct smears and concentration sedimentation methods. Among the 42 clinically suspected cases, 10 cases were positive for *C*. *philippinensis* (23.8%), while the remaining 32 cases (76.2%) were microscopically negative, but still strongly clinically suggestive of intestinal capillariasis. The stool samples of the infected patients were voluminous, yellowish to greenish, and watery to jelly-like consistency with mucus but no blood. We noticed that the microscopic findings varied according to the duration of diarrhea. The stools of patients who had complained of diarrhea for 1–4 months showed all stages of the parasite (thin, thick-shelled, embryonated, unembryonated eggs, larvae with variable sizes, and adults (**Fig 1**). In patients with diarrhea for more than 4 months, only thick-shelled eggs were detected after the formalin-ether concentration technique. Charcot–Leyden crystals of variable sizes, even within the same case, were detected in the stools of most patients. The number of these crystals increased with an increase in the severity of the clinical manifestations and the increase in the number of eggs. Regarding possible risk factors, we found that all positive cases (10) were directly involved in cleaning and eviscerating fish. After treatment, the patients were cured and returned to their normal body weights within a few months. Relapses were not observed within 3–6 months after therapy and supportive treatment.

### Evaluation of rabbit and rat anti-*C.philippinensis* hyperimmune sera

Rabbit and rat anti-*C*. *philippinensis* hyperimmune sera were evaluated using indirect ELISA against the crude *C*. *philippinensis* worm antigen and available helminth antigens in our department (crude hydatid fluid (CHF) and *Schistosoma mansoni* egg antigen). Anti-*C*. *philippinensis* hyperimmune serum displayed good reactivity with *C*. *philippinensis* crude antigen, with OD values ranging from 0.248–324, while it showed faint reactions against the available helminth antigens (crude hydatid fluid antigen and *Schistosoma mansoni* egg antigen) with OD values ranging from 0.73–0.96.

### ELISA using rabbit and rat polyclonal antibodies

Study cases were classified according to the results of the microscopic examination into the following groups: **Group I** (n = 10) included microscopically confirmed cases of intestinal capillariasis. The stool samples of patients in this group were positive for egg-to-larva or adult stages (positive control). **Group II** (n = 32) included clinically suspected patients with criteria suggestive of *C*. *philipinensis* infection. These patients had symptoms such as abdominal pain, chronic watery diarrhea, muscle wasting, cachexia, weakness, or edema, with low levels of potassium and albumin in the blood; however, the microscopic examination of their stool was negative on three consecutive days. **Group III** (n = 10) included healthy subjects (negative controls). These individuals had no symptoms, and their stool examination was negative for the parasites.

**Fig 2:**
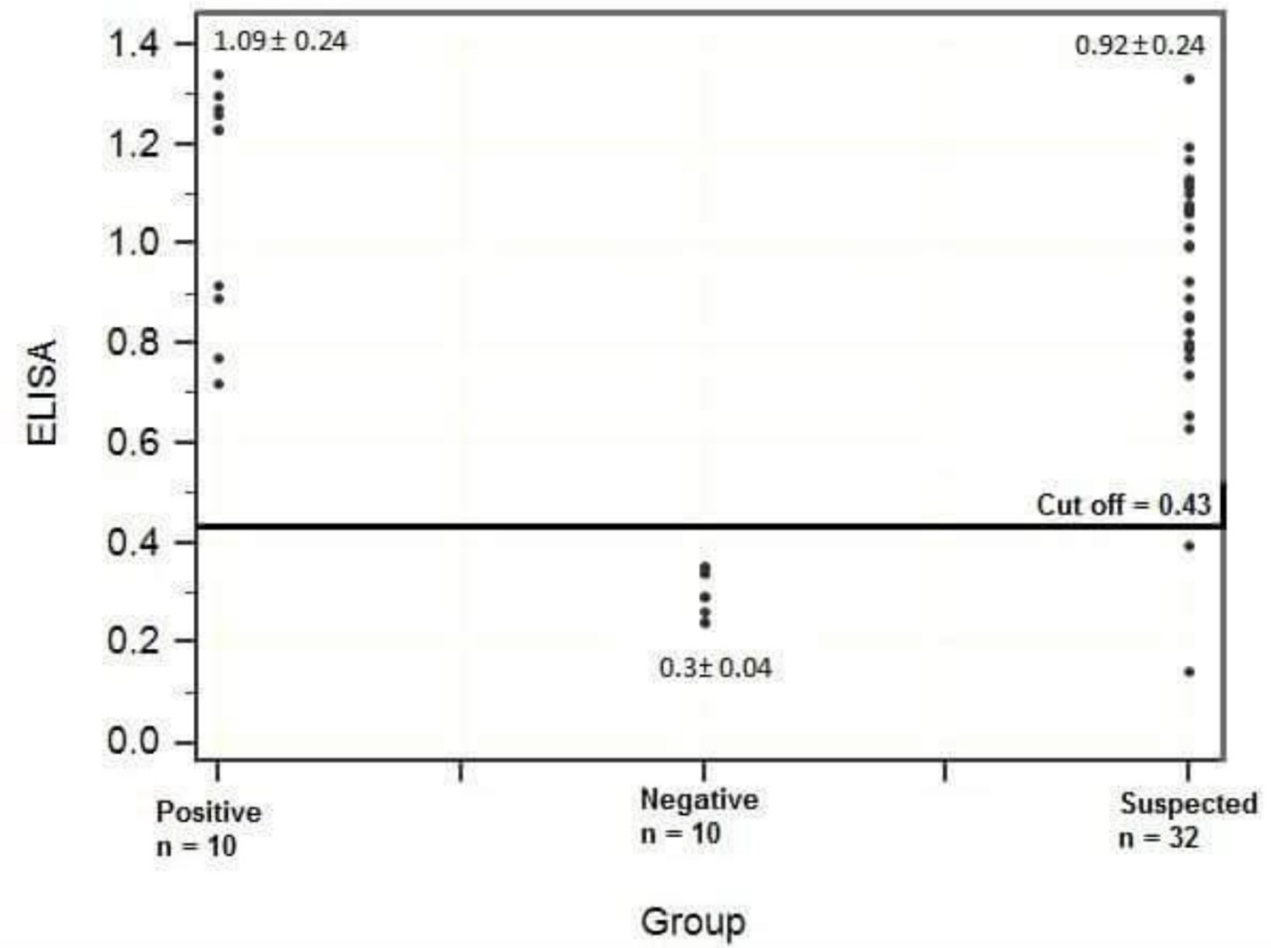
Levels of *C*. *philippinensis* coproantigen in different study groups.

Based on the calculated cutoff value (mean plus 3 SD of apparently healthy subjects), the suspected case was considered positive for coproantigen if the OD value was higher than 0.43. The assay detected all the microscopically confirmed intestinal capillariasis cases (group I). In group II (clinically suspected cases), sandwich ELISA was positive in 30 out of 32 stool elutes (93.7%), bringing the total number of ELISA-positive cases to 40 (95.2%) with 100% sensitivity (**Table 2 and Fig 2).** None of the 10 healthy subjects (group III) were positive. We observed that *C*. *philippinensis* infection detected using copro-ELISA was significantly higher (n = 38, 95%) among cases that were directly involved in cleaning and eviscerating fish (P < 0.0001).

**Table 2:** Detection of *C*. *philippinensis* coproantigen in stool eluates of different study groups.

	No. of examined cases	No. of positive cases by microscopic examination	*C*. *philippinensis* copro-antigen		
			No. of Positive cases by copro-ELISA	ELISA OD Range	mean OD
**Group I: Confirmed cases of capillariasis**	10	10	10	0.72–1.34	1.09
**Group II: Clinically suspected cases of capillariasis**	32	0	30	0.141–1.33	0.92
**Group III: Healthy subjects**	10	0	0	0.24–0.35	0.3

### Detection of *C. philippinensis* using nested PCR

**Fig 3:**
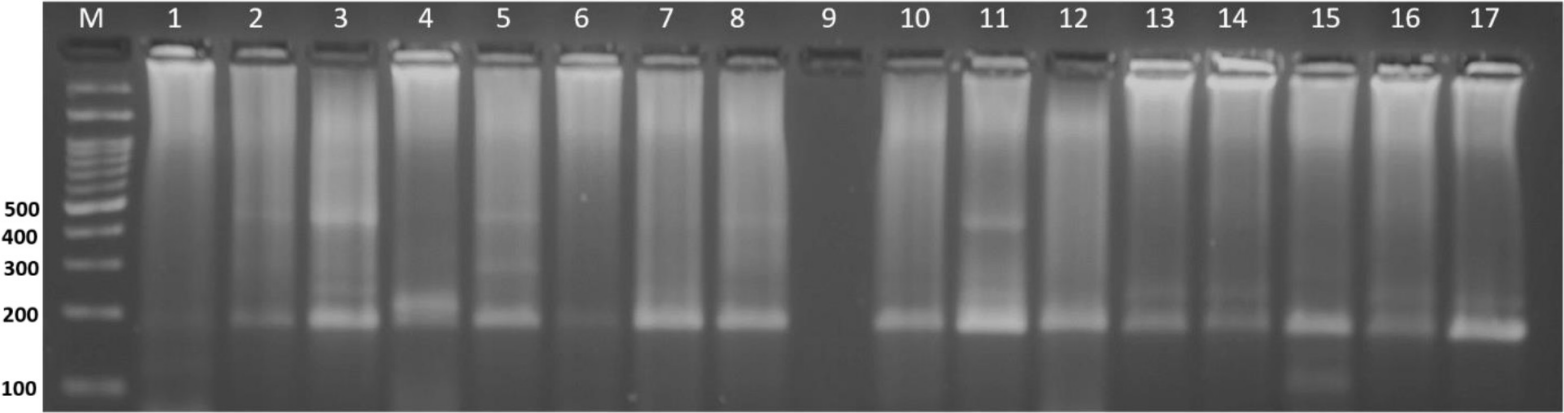
Representative nested Cp-PCR amplification products from the second PCR reaction. Positive samples show amplified products of 183 bp in lanes 1–8 and 10–16, lane 9: negative control, lane 17: positive control, and lane M: 100 bp DNA ladder.

The nested PCR reaction of the small ribosomal subunit gene in *C*. *philippinensis* amplified 235 bp and 183 bp fragments in the first and second reactions, respectively. Amplified products were detected in all cases of group I, in addition to 25 samples from group II (**Fig 3**). The total number of samples positive for *Capillaria* DNA was 35 (83.3%).

Similar to copro-ELISA, the prevalence of *C*. *philippinensis* infection was significantly higher among cases that were directly involved in cleaning and eviscerating fish; 34 (97.1%, P < 0.01).

### Sociodemographic characteristics of patients

Table 3 shows the correlation between sociodemographic characteristics and clinical data and the different test protocols. Our study reported that the majority of positive cases were middle-aged females, in the age range of 20–45 years. Most infected patients live in rural areas. In addition, all infected cases reported a history of previous involvement in cleaning and evisceration of fish, pointing to the possible transmission through contaminated hands.

**Table 3:** Correlation between sociodemographic characteristics and clinical data and the microscopic, ELISA, and PCR results among the study.

	Microscopic results			ELISA results			nPCR results		
	Positive (n = 10)	Negative (n = 32)	P-value	Positive (n = 40)	Negative (n = 2)	P-value	Positive (n = 35)	Negative (n = 7)	P-value^a^
**Sex**	** **	** **	** **	** **	** **	** **	** **	** **	** **
Male	2(20%)	7(21.8%)	0.753	7(17.5%)	2(100.0%)	0.006**	5(14.3%)	4(57.1%)	0.010*
Female	8(80%)	25(78.2%)		33(82.5%)	0(0.0%)		30(85.7%)	3(42.9%)	
**Age**	** **	** **	** **	** **	** **	** **	** **	** **	** **
< 20 years	2(20%)	8(25.0%)	0.824	9(22.5%)	1(50.0%)	0.151	8(22.8%)	2(28.6%)	0.412
20–45 years	7(70%)	19(59.4%)		26(65%)	0(0.0%)		23(65.7%)	3(42.8%)	
> 45	1(10%)	5(15.6%)		5(12.5%)	1(50%)		4(11.4%)	2(28.6%)	
**Residence**	** **	** **	** **	** **	** **	** **	** **	** **	** **
Rural	9(90.0%)	22(68.8%)	0.570	31(77.5%)	0(0.0%)	0.015*	29(82.9%)	2(28.6%)	0.003**
Urban	1 (10.0%)	10(31.3%)		9(22.5%)	2(100.0%)		6(17.1%)	5(71.4%)	
**Duration of diarrhea**	** **	** **	** **	** **	** **	** **	** **	** **	** **
1:4 months	2(20%)	23(71.9%)	0.011*	23(57.5%)	2(100.0%)	0.648	20(57.1%)	5(71.4%)	0.779
> 4months	8(80%)	9(28.1%)		17(42.5%)	0(0.0%)		15(42.9%)	2(28.6%)	
**Weight loss**	** **	** **	** **	** **	** **	** **	** **	** **	** **
Yes	10(100%)	21(65.6%)	0.081	31(77.5%)	0(0.0%)	0.015*	28(80%)	3(42.9%)	0.040*
No	0(0%)	11(34.4%)		9(22.5%)	2(100.0%)		7(20%)	4(57.1%)	
**Lower limb edema**	** **	** **	** **	** **	** **	** **	** **	** **	** **
Yes	10(100%)	9(28.1%)	<0.001**	19(47.5%)	0(0.0%)	0.188	17(48.6%)	2(28.6%)	0.332
No	0(0%)	23(71.9%)		21(52.5%)	2(100.0%)		18(51.4%)	5(71.4%)	

^a^Chi-squared and Fisher’s exact tests were performed.

Comparing the PCR results of the clinically suspected cases (Group II) with the results of copro-ELISA showed that out of 32 samples, 25 were positive for both methods, 7 (5.4%) were PCR negative, while only 2 were negative for the copro-ELISA test **(Table 4).**

**Table 4:** Comparative results of copro-ELISA and copro-PCR in the diagnosis of *C*. *philippinensis* infection among the study cases.

Coro-ELISA	Copro-PCR		Total	Sensitivity	Specificity	PPV	NPV	Accuracy
	+ve	-ve						
+ve	35	5	40 (95.5%)	100%	28.6%	87.5%	100%	88%
-ve	0	2	2 (4.7%)					
Total	35	7	42 (100%)					

## Discussion

Intestinal capillariasis is a treatable enteric parasitic infection if diagnosed properly. However, due to false diagnosis and subsequent complications, many cases have ended in death **[17]**. The diagnosis is difficult because of the atypical clinical symptoms, and parasite eggs are not always detected in stool examination. Therefore, an immunological and molecular diagnosis will provide a supportive diagnostic tool **[9, 13].**

The present study included 42 patients clinically suspected to be infected with *C*. *philippinensis* as they were suffering from manifestations similar to those reported by **Cross (1992) and Intapan et al. (2017), Attia et al. (2012), Limsrivilai et al. (2014) [1, 9, 17, 21]**. Microscopic examination showed low positivity, which could be attributed to the low parasitic load and intermittent shedding of the parasite into the stool **[21]**. This coincides with other previous studies conducted in Egypt by **Amin et al. (2011) and Ali et al. (2016) [22, 14].** Concerning the presence of Charcot-Leyden crystals, in the present work, their number correlated with the severity of the infection and the number of eggs detected, which supported the diagnosis as stated by **Cross (1992) and Saichua et al. (2008) [1, 23].**

In this study, we developed a capture ELISA using crude worm antigen anti-rat and anti-rabbit hyperimmune sera. These results indicate the superiority of the copro-ELISA to coproscopy, suggesting that the microscopic examination underestimates the real prevalence of *C*. *philippinensis* infection. Although coproantigen detection by ELISA showed very high sensitivity (100%), we had to consider the specificity of the test. Therefore, *C*. *philippinensis* hyperimmune sera were evaluated using indirect ELISA against *C*. *philippinensis* crude worm antigen and against available helminth antigens (crude hydatid fluid antigen, *Schistosoma mansoni* egg antigen), which showed the specificity of both the rabbit and rat hyperimmune sera. This is supported by previous studies by **Abdel-Rahman et al. (2019) [19],** which revealed insignificant cross-reactions between sera from *C*. *philippinensis-*infected patients and both *Schistosoma mansoni* egg antigen and hydatid cyst antigen. **El-Dib et al. (2004) [10]** observed a low level of cross-reaction between the sera of cases of *C*. *philippinensis* and *Fasciola gigantica*. However, **Lin et al. (1990) [24]** reported cross-reactions between the sera of patients with *C*. *philippinensis* and antigens prepared from *C*. *sinensis*, *Sparganum proliferum*, and *T*. *canis*. The possibility of false-positive cases could be attributed to the use of crude proteins instead of purified ones or the use of polyclonal antibodies instead of monoclonal antibodies. Therefore, our next step was to detect the infection with a more specific assay and to compare its results to the results of coproscopy and copro-ELISA.

The copro-PCR techniques that directly detect parasite DNA after amplification provide species-specific assays with a high degree of sensitivity. These molecular techniques provide some advantages over the antigen detection capture ELISA as they detect material at much lower concentrations in the stool **[25, 26]**. In addition, these copro-PCR techniques can provide diagnostic tools to overcome the limitations of microscopic examination **[27]**. In this study, nested PCR was performed to detect *Capillaria* infection among the 42 clinically suspected patients. The primers used for the nested PCR detection of *Capillaria* in our study were adopted from **El-Dib et al. (2015) [13]**. The primers were reported to be specific and sensitive to *Capillaria*-DNA because the amplified region of the ssurDNA is highly conserved for *C*. *philippinensis* [13]. In our experiments, the amplified products were not sequenced. However, to confirm the specificity of the nested PCR reaction, we tested stool samples spiked with other parasites such as *Echinococcus granulosus*, *Ascaris*, *Fasciola*, *Trichinella spiralis*, and *Giardia intestinalis* and no amplified products were obtained with the DNA of the other tested parasites.

Results of copro-PCR showed positivity in 83.3% of the suspected cases, which is higher than that reported by **Ali et al. (2016) [14],** which indicated that the prevalence rate of *Capillaria* infection was 11.6% among symptomatic cases of diarrhea. Nevertheless, the criteria employed for the selection of patients are different in our group of patients. The nested PCR detected *C*. *philippinensis* copro-DNA in all positive cases using the microscopic examination. However, five samples that were positive by ELISA were reported to be negative by nested PCR. A possible explanation is that these five samples were falsely detected by ELISA due to the use of crude protein instead of purified ones, and polyclonal antibodies instead of monoclonal antibodies.

**Ali et al. (2016) [14]** reported that the detection of capillariasis using nested PCR targeting the ssurDNA gene is a specific and accurate method that helps to determine the true prevalence and epidemiology of the disease. Considering the nested PCR as a Gold standard test, the Copro-ELISA test showed a high sensitivity rate (100%) with a positive predictive value of 87.5%, while the specificity rate was 28.6% with 100% negative predictive value. In addition, we reported a qualitative agreement between the two tests in 88% of the samples. Taken together, copro-ELISA can exclude negative cases, but copro-DNA should be employed to detect true positive cases.

Our study reported that the majority of the positive cases were females, which is in agreement with the reports by **El-Dib and Doss (2002), Attia et al. (2012), and Ali et al. (2016) [6, 17, 14].** Females are usually involved in food preparation, which increases the probability of infection. However, in some endemic countries, the disease is more prevalent among males **[28–31].** Most of the infected patients were between 20 and 45 years of age, with a mean age of 31.8. **Ali et al. (2016), Attia et al. (2012), and Cross and Bhaibulaya (1983) [14, 17, 32]** reported similar results. The majority of infected patients were from rural areas, which reflects the effect of social standards in the transmission of the disease, in accordance with **Amin et al. (2011) and El-Dib and Doss (2002) [22, 6].** All infected patients had a history of cleaning and evisceration of fish by themselves. This may have led to transmission of the infection through contaminated fingernails, as suggested by **El Dib and Doss (2002) [6].**

## Conclusion

The real number of *Capillaria* cases may far exceed those estimated using microscopy. Coproscopic examination is not a reliable method for *C*. *philippinensis* diagnosis as it missed the infection in 25 infected patients. ELISA using coproantigen is a sensitive test that identifies infection with the parasite but with low specificity. However, to ensure specificity, samples should be confirmed by the detection of parasite DNA in stool samples. Diagnosis of *Capillaria* using nested PCR offers a relatively encouraging, useful, satisfactory, and alternative method for the detection of infection in clinically suspected patients.

The epidemiological and clinical data in this study will also help us to understand the public health importance of different environmental routes of transmission and the clinical presentation of the patients. The most susceptible individuals to *C*. *philippinensis* infection are females, middle-aged people, and people of low social standards. Intestinal capillariasis needs to be considered in patients presenting with symptoms of chronic diarrhea and hypoalbuminemia. If these cases are left undiagnosed and untreated, they may suffer from lethal complications.

## Supporting information

S1 Raw images (TIF)
