# Supplementary figures and images for: Comparison of the diagnostic performance of microscopic examination, Copro-ELISA, and Copro-PCR in the diagnosis of Capillaria philippinensis infections
Source: PLoS One. 2020 Jun 17;15(6):e0234746. doi: 10.1371/journal.pone.0234746 (PMC7299379; doi:10.1371/journal.pone.0234746)

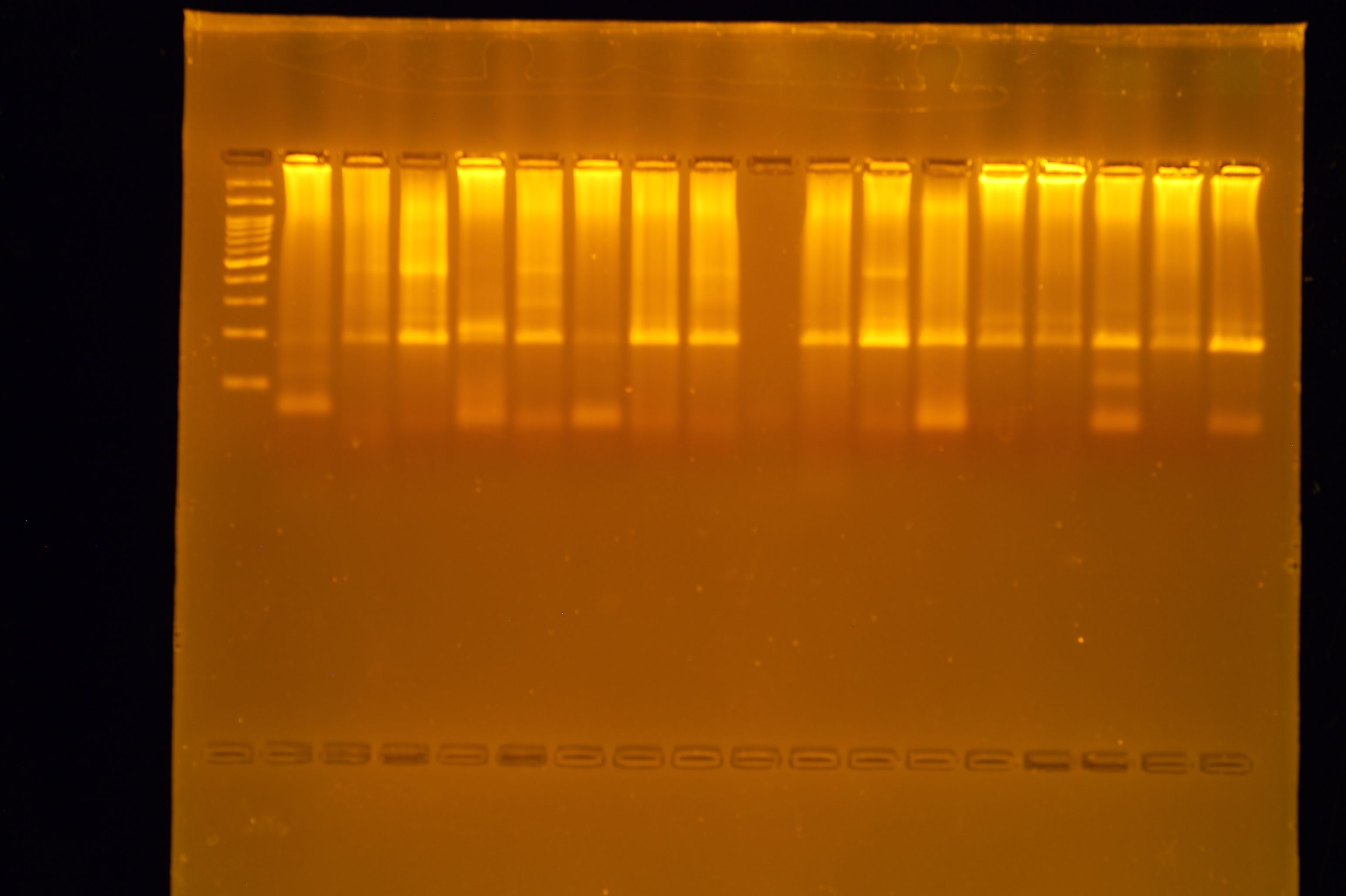

Supplement: S1 Raw images — (TIF) [file pone.0234746.s001.tif]
